# Improving Knowledge and Process for International Emergency Medicine Fellowship Applicants: A Call for a Uniform Application

**DOI:** 10.1155/2013/737391

**Published:** 2013-02-21

**Authors:** Gabrielle A. Jacquet, Jamil D. Bayram, William B. Ewen, Bhakti Hansoti, Steven Andescavage, David Price, Robert E. Suter, Alexander Vu

**Affiliations:** ^1^Department of Emergency Medicine, Johns Hopkins University School of Medicine, Baltimore, MD 21231, USA; ^2^Department of Emergency Medicine, University of Texas Southwestern, Medical Center, Dallas, TX 75390, USA; ^3^Department of Emergency Medicine, University of Chicago, Chicago, IL 60637, USA; ^4^Department of Emergency Medicine, George Washington University, Washington, DC 20052, USA; ^5^Department of Emergency Medicine, Gwinnett Medical Center, Lawrenceville, GA 30096, USA

## Abstract

*Background*. There are currently 34 International Emergency Medicine (IEM) fellowship programs. Applicants and programs are increasing in number and diversity. Without a standardized application, applicants have a difficulty approaching programs in an informed and an organized method; a streamlined application system is necessary. *Objectives*. To measure fellows' knowledge of their programs' curricula prior to starting fellowship and to determine what percent of fellows and program directors would support a universal application system. *Methods*. A focus group of program directors, recent, and current fellows convened to determine the most important features of an IEM fellowship application process. A survey was administered electronically to a convenience sample of 78 participants from 34 programs. Respondents included fellowship directors, fellows, and recent graduates. *Results*. Most fellows (70%) did not know their program's curriculum prior to starting fellowship. The majority of program directors and fellows support a uniform application service (81% and 67%, resp.) and deadline (85% for both). A minority of program directors (35%) and fellows (30%) support a formal match. *Conclusions*. Program directors and fellows support a uniform application service and deadline, but not a formalized match. Forums for disseminating IEM fellowship information and for administering a uniform application service and deadline are currently in development to improve the process.

## 1. Introduction

**Figure 1:**
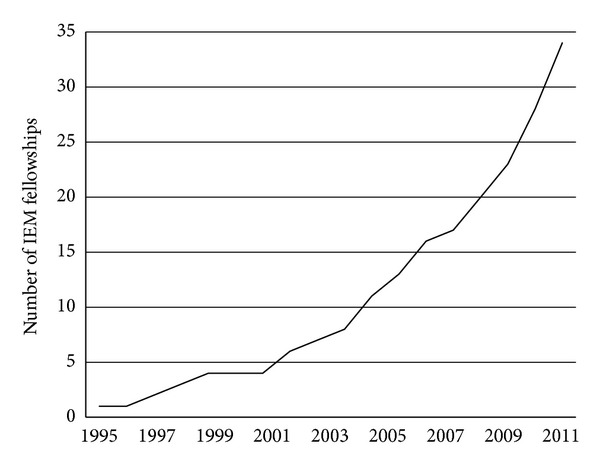
Cumulative number of IEM fellowships over time.

The field of emergency medicine (EM) as a recognized specialty continues to grow worldwide. As of 2010, over 60 nations have officially recognized EM as a specialty [1]. The global trend towards recognition of EM as a core specialty has created opportunities for emergency physicians to assess and develop fledgling academic EM practices on the world stage. Every year the number and diversity of International Emergency Medicine (IEM) fellowships in the USA multiply exponentially [2]. Since the first IEM fellowship program began in 1995, at University of Chicago at Illinois, the number of IEM fellowships has steadily risen to 34 currently active fellowship programs (Figure 1).

There are now even more specialized IEM fellowships focusing on pediatric IEM, global health leadership, and even international emergency ultrasound education. The increasing availability and diversity of IEM training programs has created a need for a more organized way to disseminate information about program curriculum and characteristics to prospective applicants. Core curriculums have been proposed for many other fellowships: Medical Toxicology [3, 4], Emergency Medical Services [5–7], Administration [8], Emergency Ultrasound [9], Pediatric Emergency Medicine [10], Disaster Medicine [11], and Education [12]. Similarly, core curriculum components of IEM fellowships have been proposed [13–15]; however, at this time, program curricula are not standardized and do not consistently offer or require these components to be completed during the fellowship. 

Other fellowships, such as subspecialties within the fields of internal medicine and surgery, have turned to the National Resident Matching Program (NRMP) [16] or another similar system for matching fellow candidates in order to make the application process more efficient. Within the field of emergency medicine, some fellowships such as emergency ultrasound and emergency critical care have recently started using universal websites with their own comprehensive uniform application systems [17, 18]. Information on these websites can be updated easily and frequently by program directors or administrative staff. 

Historically, other websites have attempted to provide information to applicants regarding program focus, current clinical and research projects, application requirements, and contact information. Much to the dismay of applicants, these websites typically have outdated contact information and other vital statistics. To the best of the authors' knowledge, there is currently no accurate up-to-date database to inform applicants about the various fellowship programs available. In addition, there is no uniform application process or universal deadline. Applicants often do not fully understand the various options available to them and ultimately make uninformed decisions. Without a uniform application deadline, many applicants are offered positions at certain programs several months before other programs have even started interviewing. Each year, several fellowship programs are left unfilled for preventable reasons such as lack of knowledge among applicants and applicants receiving offers that expire while they are waiting for more desirable offers. This survey sought to determine the needs of IEM fellowship directors and applicants in order to improve the application process for IEM subspecialty training programs and to explore potential improvements. 

## 2. Methods

A total of 34 current IEM fellowship programs were identified through a search of fellowship databases hosted by the American Academy of Emergency Medicine (AAEM) [19], Society for Academic Emergency Medicine (SAEM) [20], and Emergency Medicine Residents Association (EMRA) [21] and an Internet search using the terms “International Emergency Medicine Fellowship.” Program directors and current fellows were contacted through email addresses provided on the AAEM, SAEM, and EMRA websites. When website contact information was out of date or invalid, personal contacts of the authors were used to obtain correct contact information. Many program directors also provided the names and contact information of their current fellows and recent fellowship graduates in a modified snowball method. Institutional review board approval was obtained from the host institution. 

A focus group of interested program directors, recent, and current fellows from five academic institutions convened to determine the most important features of an IEM fellowship application process. The survey had faced was validity because it designed using the results of this well-represented, diverse focus group. Questions were asked about prior knowledge of their program's curriculum, as were opinions about ideas to improve the application process. Since the programs are extremely different from each other, an attempt was made to quantify this dissimilarity. Respondents were also surveyed about other aspects of their IEM fellowship programs including clinical hours, salary, and benefits.

In November 2011, the electronic survey was administered to 78 participants from the 34 fellowship programs; current IEM fellowship directors, current fellows, and recent graduates (within the past 3 years). Informed consent was directly obtained from all survey participants. In cases of nonresponse, followup messages were sent out electronically. Three out of 34 programs (9%) reported that they currently have no fellow due to inability to fill the position and/or lack of funding. The results from these 3 IEM fellowship programs were included in the analysis. Another 2 programs that had previously offered an IEM fellowship have closed for the same reasons. The results from these 2 programs were not included since they are no longer operational. All statistical analysis was performed using STATA 11 software (College Station, TX, USA).

## 3. Results

Response rates were 68.4% for program directors (*n* = 26) and 67.5% for current fellows and recent fellowship graduates (*n* = 27). For programs with codirectors, surveys were completed and returned by each program director individually. In the process of attempting to contact program directors and fellows using posted information on U.S. Emergency Medicine organization websites such as SAEM, EMRA, AAEM, a substantial amount of the contact information previously posted on other fellowship databases was noted to be inaccurate or out of date.

The first part of the survey assessed the availability of information and the level of knowledge available to applicants prior to starting the fellowship. Current fellows and fellowship graduates were asked how well they understood their fellowship curriculum before they started and why they chose their fellowship program. The majority of fellows stated that they did not know their program's curriculum very well before starting the fellowship (Table 1). 

The proportion of fellows who chose their program based on the fellowship's curriculum or its area of focus was the same as the proportion of those who made their choice based on the institution's name or reputation. 

The majority of current and recent fellows and program directors support transitioning to a uniform application service and a uniform application deadline, as shown in Table 2. 

However, at this time, only one-third of program directors and fellows support the use of a formalized match process such as NRMP.

### 3.1. Additional Findings

Respondents were also surveyed about other aspects of their IEM fellowship programs. Most IEM fellowship programs take 1-2 new fellows per year (74%) and are established as 2 years in length (73%). Fellowship directors typically reported that the length of program (1 versus 2 years) depended on the fellow's concurrent enrollment in a Masters Degree program. The mean reported annual salary for first year fellows is $85,593.75 (SD $21,319) with a range from $55,000 to $140,000. Many other respondents reported using PGY-adjusted salary for their individual institution, but this amount was not specified. The mean annual clinical hours obligation is 780 (SD 75.8) with a range of 650 to 850. Respondents also answered questions regarding availability of various services, benefits, and travel funding to fellows, as shown in Table 3. 

Fellows were also asked how much time they spent abroad over the course of their entire fellowship. The majority of fellows (62%) spent a total of 3–6 months abroad, while 23% spent more than 6 months abroad and 15% spent less than 3 months abroad. Fellows were also surveyed about their future career aspirations when entering fellowship. The majority of fellows (72%) stated that they planned to pursue a faculty position at an academic institution with international projects. 

## 4. Discussion

The results of the survey reveal a great deficit in knowledge of applicants. With the majority of fellows only understanding their program's curriculum *somewhat *or* not at all* prior to choosing their IEM fellowship, it is imperative that an unbiased and up-to-date database of IEM fellowship program information be developed and maintained. Similar to the FREIDA online database [22], which is supported by the American Medical Association, this website would be an exhaustive resource updated annually by fellowship directors or their administrative staff. Several websites have attempted this endeavor in the past with information on all fellowship programs, but much of the information is invalid just a few years after their original creation. With the support of the majority of IEM fellowship directors, recent graduates, and current fellows, change can and should be implemented to promote informed decisions and an efficient application process for all parties involved. 

This survey also supports the institution of a uniform application service and deadline. Based on this new information, the IEM Fellowship Consortium is currently designing an IEM Fellowship website and application system that will ensure informed decisions, eliminate superfluous efforts, and facilitate better matches between programs and fellows. The survey results also suggest that, at this time, most program directors and fellows do not support a formalized match process. If this changes in the future, the standardized application platform may be reconfigured to serve in this capacity.

*Limitations.* This study was limited by a small sample size and a lower response rate of current and recent fellows. In addition, some respondents only answered some of the questions and therefore their surveys were incomplete. Several programs were unable to provide current fellow input due to lack of enrollment.

## 5. Conclusions

A profound knowledge gap exists between fellowship applicants and program curricula. The majority of IEM fellows and program directors support the use of a standardized application process and deadline. This will enable the IEM community to effect change in our recruitment strategy and application process. Accurate and easily accessible information about each program can ensure the best fit for both candidates and IEM fellowship programs and greater career satisfaction for IEM fellows.

## Figures and Tables

**Table 1:** Fellows' prior knowledge of curriculum and reasons for choosing program.

	Response choice	Percent who replied
How well did you know your program's curriculum before you started?	Not at all	30%
	Somewhat	40%
	Very well	30%

Why did you choose your program?	Name/reputation of institution	41%
	Curriculum	41%
	Countries/regions of interest	41%
	Program director	32%
	Salary	18%
	Other	14%

**Table 2:** Program director and fellow support of a uniform application system.

	Program directors *n* = 26	Current and recent fellows *n* = 27
Support a uniform application service	81%	67%
Support a uniform application deadline	85%	85%
Support a formalized match process such as NRMP	35%	30%

**Table 3:** Other benefits offered by fellowship programs.

	Program directors (*n* = 20)	Recent/current fellows (*n* = 18)
Is CME covered?	85%	89%
Do you have access to a statistician?	60%	56%
Are the program project expenses covered?	74%	83%
Are the personal project expenses covered?	80%	56%
